# T_1ρ_-Mapping for Musculoskeletal Pain Diagnosis: Case Series of Variation of Water Bound Glycosaminoglycans Quantification before and after Fascial Manipulation^®^ in Subjects with Elbow Pain

**DOI:** 10.3390/ijerph17030708

**Published:** 2020-01-22

**Authors:** Rajiv G. Menon, Stephen F. Oswald, Preeti Raghavan, Ravinder R. Regatte, Antonio Stecco

**Affiliations:** 1 Bernard and Irene Schwartz Center for Biomedical Imaging, New York University School of Medicine, New York, NY 10016, USA; Rajiv.Menon@nyulangone.org (R.G.M.); Ravinder.Regatte@nyulangone.org (R.R.R.); 2 Private Practice, New York, NY 10011, USA; drstephenoswald@gmail.com; 3 Departments of Physical Medicine and Rehabilitation and Neurology, Johns Hopkins University School of Medicine, Baltimore, MD 21205, USA; praghav3@jhmi.edu; 4 Rusk Rehabilitation, New York University School of Medicine, New York, NY 10016, USA

**Keywords:** T_1ρ_, fascial manipulation, epicondylitis

## Abstract

Diagnosis and management of musculoskeletal pain is a major clinical challenge. Following this need, the first aim of our study was to provide an innovative magnetic resonance technique called T_1ρ_ to quantify possible alterations in elbow pain, a common musculoskeletal pain syndrome that has not a clear etiology. Five patients were recruited presenting chronic elbow pain (>3 months), with an age between 30 and 70 years old. Patients underwent two T_1ρ_-mapping evaluations, one before and one after the series of Fascial Manipulation^®^ (FM) treatments. After the first MRI evaluation, a Disability of the Arm, Shoulder and Hand (DASH) questionnaire was administered to quantify the symptoms and pain intensity. Patients then received three sessions of FM, once a week for 40 min each. A statistically significant difference was found between bound and unbound water concentration before and after FM treatment. Our preliminary data suggest that the application of the manual method seems to decrease the concentration of unbound water inside the deep fascia in the most chronic patients. This could explain the change in viscosity perceived by many practitioners as well as the decrease of symptoms due to the restoration of the normal property of the loose connective tissue. Being able to identify an altered deep fascial area may better guide therapies, contributing to a more nuanced view of the mechanisms of pain.

## 1. Introduction

Diagnosis and management of musculoskeletal pain is a major clinical challenge [1]. Non-malignant musculoskeletal pain is the most common clinical symptom that causes patients to seek medical attention and is a major cause of disability in the world [2]. In a large study, 842 patients presenting to the emergency department with moderate to severe pain found that 74% of patients continued to experience pain of moderate to severe intensity at discharge [3]. Hence, we need to strengthen our understanding of the symptoms and the related mechanisms underlying musculoskeletal pain. Fundamental knowledge of nociception from deep somatic structures has been studied and defined in animals but the translation into clinical (human) sciences is still lacking [1]. Even if most clinicians consider chronic pain to be typically due to ongoing peripheral nociceptive input, there is not a single chronic pain state where any radiographic, surgical, or pathological description of peripheral nociceptive damage has been reproducibly shown to be related to the presence or severity of pain [4]. Many studies have reported pain in the absence of tissue damage or with likely pathophysiological cause. Further, it is usually not possible to distinguish their experience from that due to soft-tissue damage [5]. Even muscle biopsy studies had not conclusively shown specific abnormalities for chronic pain syndromes [6,7]. Arendt-Nielsen et al. [1] state that, in humans, little information is available on the peripheral neuronal correlate of muscle nociceptor activation and, therefore, quantitative non-invasive techniques are needed for translational pain research. Following this need, we designed our study with an innovative technique to quantify possible alterations presented in symptomatic elbow. Shawan et al. [7] explain that the location and quality of elbow pain can be generally localized in the anterior, medial, lateral, or posterior region. Lateral and medial elbow pain are two of the more common diagnoses and often occur as a result of occupational activities but, unfortunately, determining the underlying etiology of elbow pain can be difficult. Even if MRI is the preferred imaging modality for chronic elbow pain due to its excellent soft-tissue contrast [8,9], most conditions that cause chronic elbow pathology have only a clinically based diagnosis [7].

Recently a new MR-technique using T_1ρ_ mapping to image water-bounded content in upper limb muscles was proposed [10]. T_1ρ_ mapping is sensitive to low-frequency interactions [11], related to the chemical exchange between extracellular water (unbound water) and macromolecules (bound water) [12]. T_1ρ_ mapping allows an indirect quantification of glycosaminoglycan (GAG) content, and has been used to quantify proteoglycan content in cartilage [12], muscle [13], and intervertebral discs [14].

Arendt-Nielsen et al. [1] affirm that developing an understanding with novel mechanism-based therapies to treat musculoskeletal pain would address a major unmet clinical need and will have significant clinical, economic, and societal benefits. With this purpose, the aim of this study was to treat patients suffering from elbow pain using Fascial Manipulation^®^ (FM), a manual technique that modifies the fascia [15,16] leading to change the excitability threshold of peripheral receptors within fascia [17,18]. Knowing the existence of a high concentration of free nerves endings and receptors within the fascia [17,18], this tissue has therefore obtained a new important role in pain generation, attracting many researchers [1].

## 2. Material and Methods

In this case series, five patients were prospectively recruited presenting chronic elbow pain (>3 months), with an age between 30 and 70 years old, presenting symptoms prevalent in the lateral region of the elbow which appeared without apparent trauma. Exclusion criteria for the study were subjects with previous elbow fractures, surgery, or concomitant neurological or rheumatological pathologies that could have altered the normal anatomy. This study was approved by New York University Langone Health’s institutional review board (IRB) and was health insurance portability and accountability act (HIPAA) compliant (S16-01317). This was a prospective, non-randomized imaging study to determine intramuscular GAG content using proton T_1ρ_ relaxation mapping. All subjects gave written informed consent after explanation of the study and the protocol, as per the IRB guidelines.

Patients underwent two T_1ρ_ mapping evaluations, one before and one after the series of FM treatments. T_1ρ_-MRI was performed at the Bernard and Irene Schwartz Center for Biomedical Imaging, New York University School of Medicine. All imaging scans were done on a clinical 3T MRI scanner (Prisma, Siemens Healthineers, Erlangen, Germany). The subjects were instructed to lie in a prone position during the MRI imaging session. An 8-coil flexible receive array coil was wrapped around the arm. A 3D-turbo-FLASH (fast, low-angle shot) MRI sequence with a customized T_1ρ_-preparation module was used to enable varying spin lock durations (TSL). The sequence parameters included FOV = 130 mm, matrix size = 256 × 64 × 64, TR = 1500 ms, resolution = 0.5 mm × 2 mm × 2 mm, spin lock frequency = 500 Hz, 10-TSL durations = 2, 4, 6, 8, 10, 15, 25, 35, 45, 55 ms acquisition duration = ~10 min. The MRI body coil was used for transmission, and vendor-supplied flexible receive array coils (8 coil elements each) were wrapped around the affected arm for imaging. The defined landmarks were adopted in order to standardize the location of the regions of interest as to where on the limb the MR imaging was performed. Seven sites were selected in the forearm, equidistant from each other, from the lateral epicondyles to the musculotendon junction of the common extensor tendon. The advantage of T_1ρ_ is that it can quantify indirectly the concentration of the GAG in the musculoskeletal system. T_1ρ_ mapping allows the quantification of the amount of water that is bounded or not with GAG. GAG includes hyaluronan (HA) and proteoglycans. HA is normally considered a lubricant that allows the gliding between interfaces such as cartilage to cartilage, epimysium to deep fascia, and between individual layers of the deep fascia [19]. In specific conditions, however, when it is spread on a surface at high concentration, HA can aggregate through non-covalent interactions like those characteristic of van der Waals and hydrophobic forces, dramatically increasing the viscosity of the surrounding loose connective tissue [20].

**Figure 1:**
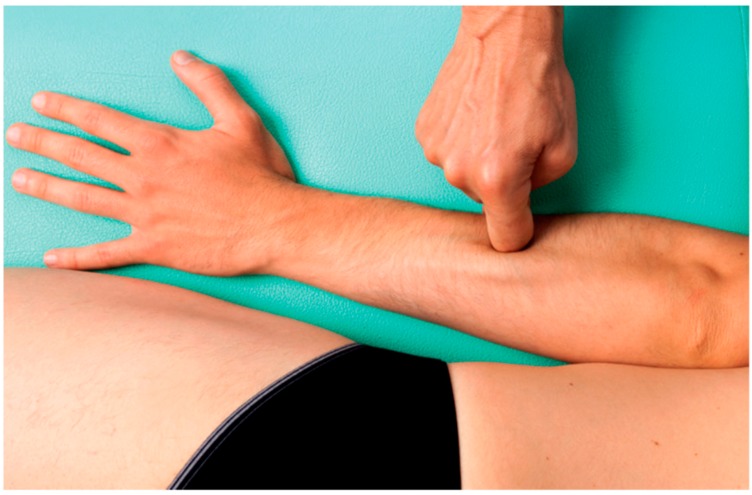
Treatment over the center of coordination ER-CA (see Figure 2).

**Figure 2:**
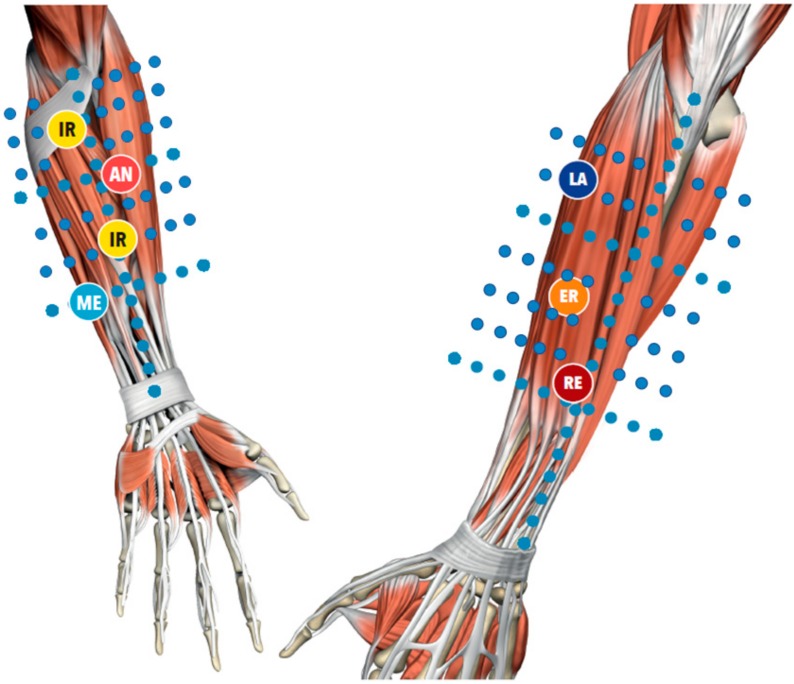
Location of the center of coordination. AN = antemotion; RE = retromotion; ME = mediomotion; LA = lateromotion; IR = intrarotation; ER = extrarotation. The blue dots represent the slides were coronal MRI were made.

After the pre-treatment T1ρ-mapping evaluation, a DASH questionnaire was administered to quantify symptoms and pain intensity. Patients then received three sessions of FM, once a week for 40 min each, after which the DASH questionnaire was administered again. FM is a manual technique (Figure 1) that implies a modern biomedical approach to the illness, thanks to the knowledge of the human fascial system, but, at the same time, uses an individual approach to the patient. During the clinical history, the segments in dysfunction are identified with an emphasis on the chronology. This permits the development of a treatment hypothesis based on the current symptomatology of patients and previous musculoskeletal events, which may be causing compensations, leading to pain even in distant sites. The selection of points, defined as centers of coordination (CC) and centers of fusion (CFs) (Figure 2), is guided by an assessment chart [21,22]. The chart integrates information collected through movement verifications, patient pain ratings, including any radiation, and, most importantly, the presence of “densification” (soft-tissue stiffness), obtained through palpation verification. Additional guidance for the selection of points includes avoiding the patient’s excessively painful areas where inflammation, lesions, or even fractures could be present, making this deep friction a safe treatment.

## 3. Statistics

Data analysis was performed with the SPSS software, version 25, (SPSS Inc., Chicago, IL, USA). Efficacy of the treatment, assessed through the DASH score, was treated as an ordinal variable using nonparametric statistical test (Wilcoxon–Mann–Whitney test). The mean values and standard deviations in the manually drawn region of interests (ROIs) were calculated across the patients. Wilcoxon–Mann–Whitney test was also used for comparing the pre- vs. post-treatment conditions in patients. A *p*-value of less than 0.05 was used for analysis as the threshold to reject the null hypothesis.

## 4. Results

Five patients were recruited with an average age of 57.4 (SD 7.92): two males and three females. The average time since the onset of the symptoms was 30 months (SD 18.97). No side effects were reported during or after treatment. Approximately one to two months following the treatment, the patients were scanned again using the same T_1ρ_-mapping protocol. For post-treatment imaging, only four of the five treated subjects were scanned, due to a subsequent dental implant in one patient. Only the subjects having pre- and post-treatment MRI scans were included in the pre- vs. post-treatment analysis.

The DASH questionnaire had a mean value of 49.95 (SD 19.93), DASH work 43.64 (SD 23.50), and DASH sport 65.07 (SD 34.95) before treatment. The DASH score ranged from 0 (no symptoms) to 100 (unable to perform any activities). After treatment, the questionnaire presented a mean value of 32.80 (SD 7.03), DASH work 32.03 (SD 13.84), and a DASH sport of 35.60 (SD 11.65) with a statistically significant difference in 21 items over 30 and four items over five in work and sport DASH score (Table 1, Table 2, Table 3 and Table 4).

**Figure 3:**
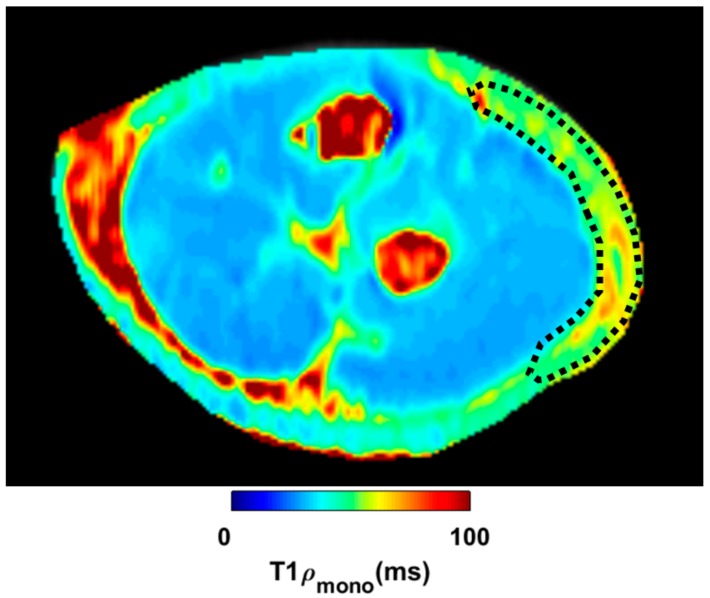
Region of interest (ROI) in one slide post-treatment. The ROI is the black line located below the skin at the level of the deep fascia in this transversal section of the forearm.

**Figure 4:**
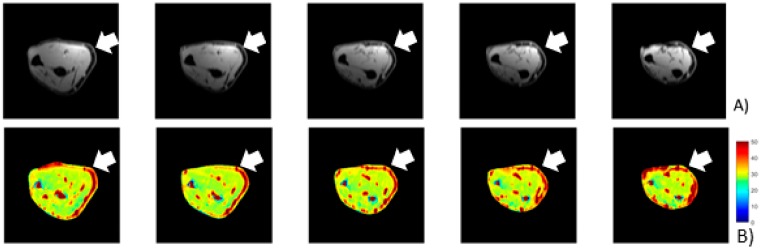
(**A**) Pretreatment of T1 MRI where the thickness of the brachial fascia is evident. The fascia is the black ring below the skin identified by the arrow. (**B**) Pretreatment T_1ρ_-mapping where the higher concentration of unbounded water is evident (water that it is not linked with any glycosaminoglycan or hyaluronan) within the brachial fascia in the symptomatic side (white arrow).

MATLAB software was used to evaluate the quantity of each single-color pixel in the seven MRI slices over a pre-defined ROI corresponding to the lateral region of the elbow, the anatomical area where most of the symptoms were referred (Figure 3). The selection of the ROI area was arbitrary with the intent to isolate the deep fascia: a “second skin” that appears in the circumference of each coronal section. A major red region (high concentration of unbounded water) was identified at the level of the antebrachial fascia (deep fascia) in the pretreatment T_1ρ_ sequence (Figure 4a). Some red spots were also present in the middle of the forearm, corresponding to intermuscular septa and vessels. A statistically significant difference was found between bound and unbound water concentration, before and after FM treatment (Table 5). The slices shown in Figure 4a,b correspond to approximately similar slice positions in the affected arm.

**Figure 5:**
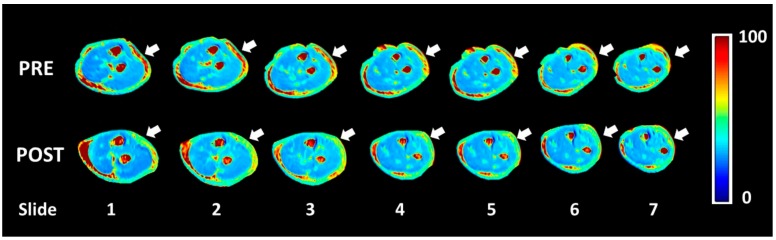
Pre- and post-T_1ρ_-mapping of brachial fascia (in ms). The white arrows show the areas that were considered the most symptomatic from the patient. It can be seen a change of the color from red (unbound water) in the pre-treatment imaging to green-blue (bound water that works as a lubricant) in the post-treatment imaging.

Most of the patients presented a statistically significant difference in T_1ρ_ relaxation time between pre- and post-treatment conditions in the selected ROI (Table 5). Unfortunately, only in the most severe patients there was a clear decrease of the unbounded water. In patient p_4, following the Fascial Manipulation treatment, the mean T_1ρ_ relaxation time in the ROI decreased from 72.16 ms (SD 2.86) to 53.36 ms (SD 6.72) (Figure 5). While there were smaller focal reductions in the unbound water in the less chronic patients, the overall ROIs showed a net increase in the T_1ρ_ values. It may be related to a general biomechanical change of all the forearm region following the treatment of CCs and CFs that, even if opposite or far away from the lateral region of the elbow, they may have affected the physiology of the evaluated area. We also have to take into consideration that minor symptoms should also be less extended or maybe localized proximal or distal from the lateral epicondyles, limiting the ability of the pre-defined ROI to collect those alterations. If so, the increase of unbounded water, detected from our ROI, could be considered a redistribution or new organization of the remaining unbounded water in all the body segments.

## 5. Discussion

For the last two to three decades, pain research has mainly focused on the sensitization of nociceptors in the periphery [1] without clearly defining the cause. Cowman et al. [23] has proved that increases in HA concentration, the chief component of the GAG, can trigger a self-aggregation, generating a dramatic increase in the viscosity in the extracellular matrix. This alteration can affect the polymodal nociceptors [24] included in the deep fascia [17,18], decreasing their threshold of activation [25]. This means that painful signals can easily be generated from abnormal peripheral tissues (for example, stiff fascia) and transmitted, by the dorsal horn pain transmission neurons, to the brain [26].

Being able to identify an altered deep fascial region may better guide therapies, contributing to a more nuanced view of the mechanisms of pain rather than simply knowing “diagnoses” from which the patient is suffering [4], and using this as the basis for treatment. In a recent survey [3], 294 patients were followed prospectively for a year. Of these patients, 62% suffered musculoskeletal pain. Despite a year of treatment, 95% of the patients were still suffering from moderate to severe pain. Arendt-Nielsen et al. [1] affirm that this data may very well represent the clinical situation observed by many general practitioners. Elbow pain, epicondylitis, epitrochleitis, etc. could be examples of regional pain conditions characterized by localized tenderness and pain which are caused by altered fascia. A correct diagnosis will allow defining a better treatment, decreasing not only the patients’ sufferance but also the cost for the entire society.

FM, even if it shares some similarities with other techniques, presents a deeply different rationality and clinical approach. While deep friction seen in FM can be compared to other techniques, the reasoning behind the choice of points treated presents a major difference. The points are selected after a specific assessment process involving clinical history, taking a clinical examination of specific movements and palpatory verifications with the aim to identify stiff CCs and CFs [21] mostly outside the painful site.

MRI imaging already played a critical role in evaluating elbow pain [27], but now, with the T_1ρ_ mapping technique, it can become critical to define pain etiology. The process, that requires only a few minutes more than a normal evaluation, provides data and imaging that can guide the clinician in identifying the origin of the problem and the areas where treatment should be applied.

Our pilot study has shown that a high concentration of unbounded water was present even more in outside regions from the symptomatic areas, regions where FM treatment is normally performed (Figure 2). The information collected from the T_1ρ-_mapping can now give rationality to many techniques that approach the symptoms by treating areas distant from the symptoms’ sites. This may explain why a moderate relation between the DASH score and the concentration of bound water was found. At the opposite end, a high concentration of unbound water was highly related to elevated DASH score and symptoms. The statistically significant difference in T_1ρ_ relaxation time between pre- and post-treatment in patient one and two still proves a change of the quality of the tissue in the lateral elbow, even if the concentration of the bound water is not increased locally. A single and predefined ROI does not permit to exclude improvements that occurred in other important biomechanical areas.

It was shown that hyaluronidase is able to decrease high HA concentration in patients suffering from muscle stiffness [10]. Interestingly, although with a lower scale, T_1ρ_ values showed elevated levels of high unbound water before HA treatment, similar to the severe case of elbow pain in P_4.

A similar result was also found after treatment where hyaluronidase injection decreased the amount of unbounded and increased the bounded water [10]. The authors suggest that hyaluronidase, with its capacity to decrease the concentration of HA, was able to deplete the area from an excess amount of HA that was not bound with water and so was not in its physical condition to operate as a lubricant.

These preliminary data are supported by in vitro studies that confirm the capacity of HA to self-aggregate and consequently increase its viscosity [23]. In our in vivo studies, we speculate that increased T_1ρ_ values reflect greater deposits of unbound GAG and HA and so are not able to express their lubricant property within the deep fascia. This peculiar state of isolated self-aggregate GAG or HA without water may explain the stiffness [15,22] as well the pain perceived probably due to the irritation of the free nerve ending and receptor within the deep fascia that do not have a proper gliding between its layers or with the underlying muscle [28].

Intervention with the aim of reducing the imbalance between self-aggregate HA/GAG (unbounded) and water aggregate HA/GAG (bounded) represents a potential therapeutic target. FM applied over CCs and CFs seems to hydrolyze the excess amount of self-aggregate GAG/HA reducing the viscoelasticity of the ECM in the affected regions and allowing the fasciacyte [29] to produce the correct quantity and quality of HA that will bound water for generating an appropriate lubricant solution required within the fascial layers.

These preliminary data suggest that, because alterations were shown in five or even seven coronal sections located between the epicondyles and the musculotendinous junction of the forearm, treatment should be performed wider than usual, including altered areas that are far from the symptoms but important from a biomechanical point of view.

This study had several limitations. The patient cohort used in this study is small. This study received an institutional review board (IRB) compliancy for imaging with new MRI sequences under development, limiting the recruitment at no more than five participants. For the ROI was arbitrary selected the lateral part of the elbow, as the most common region of symptoms, without taking into consideration the great variety of symptomatology that patients could have, as well the low correlation between the area of symptoms and origin of the problem. Previous studies have demonstrated, through elastrosonography [15] or intra/inter reliability palpation verification tests [22], that CCs and CFs, even far away from the regions of the symptoms, can affect the biomechanics of proximal or distal articulations.

No control group as well follow-up was present to prove the short- and long-lasting effects. This case series does not permit to conclude cause and effect relationships, but due to the chronicity of the patients, future clinical trials should be conducted.

## 6. Conclusions

Our understanding of pain has greatly improved over the last few decades [30], even if many questions are still unanswered. There is a general agreement in applying an early and aggressive treatment to prevent chronicity of pain when it is possible. There is also a general agreement on the need for an evidence-based, individualized treatment strategy in patients with chronic pain disorders [31]. Patient P_4 was the most chronic patient with the biggest improvement following treatment. The *p*-value of 0.00058 of the pre- vs. post-treatment supports our hypothesis of quantifying the increase in the bound water with FM treatments. Although focal improvements in small focal areas were noted in the pre- vs. post-treatment, the less chronic patients did not show significant improvement using MR-quantification.

The goal of this case series was not to explain the full mechanisms of pain perception but to better investigate the application of T_1ρ_-mapping in the detection of musculoskeletal alteration in patients who have mainly received clinical diagnosis without any imaging-based causal evidence. This manuscript suggests that we might finally have made some science-based progress with respect to etiology in pain fields in order to implement our clinical practice. Furthermore, T_1ρ_ mapping is sensitive to the chemical exchange of large macromolecules such as HA and GAG with protons in bulk water, making it a useful tool to quantify their content before and after treatment. These preliminary data have shown an excess amount of unbounded water in the symptomatic areas as well in surrounding areas, such as CCs and CFs, that have shown to decrease after treatment. These results suggest that using T_1ρ_ imaging shows significant potential to enhance the management of musculoskeletal pain, which is a very common syndrome to treat. However, more studies have to be done to gain deeper insights into the management of pain.

## Figures and Tables

**Table 1:** DASH score of the mean value of items 1–15.

ITEMS	1	2	3	4	5	6	7	8	9	10	11	12	13	14	15
**PRE**	2.29	1.71	2.00	1.57	2.00	2.00	2.00	1.86	1.86	2.00	2.43	2.57	1.86	3.00	2.43
**POST**	1.71	1.14	1.43	1.14	1.29	1.29	1.14	1.29	1.29	1.43	1.29	1.43	1.14	1.86	1.43
***p*-value**	0.165	0.15	0.549	0.002	0.004	0.068	0.016	0.010	0.016	0.172	0.007	0.051	0.000	0.504	0.269

**Table 2:** DASH score of the mean value of items 15–30.

ITEMS	16	17	18	19	20	21	22	23	24	25	26	27	28	29	30
**PRE**	1.71	1.00	2.57	2.43	1.57	1.00	1.29	1.57	2.14	2.71	1.86	2.57	2.29	2.00	2.86
**POST**	1.00	1.00	1.71	1.14	1.14	1.00	1.29	1.29	1.43	1.57	1.43	1.57	1.43	1.29	2.00
***p*-value**	0.000	0.001	0.023	0.003	0.000	0.001	0.015	0.010	0.040	0.255	0.042	0.199	0.119	0.013	0.736

**Table 3:** DASH work and DASH sport score.

WORK				
ITEMS	1	2	3	4
**PRE**	1.71	2.00	1.86	1.57
**POST**	1.29	1.29	1.43	1.29
***p*-value**	0.046	0.168	0.030	0.010

**Table 4:** DASH work and DASH sport score.

SPORT				
ITEMS	1	2	3	4
**PRE**	2.71	2.86	2.57	2.43
**POST**	1.57	1.43	1.57	1.29
***p*-value**	0.36	0.36	0.65	0.11

**Table 5:** Mean T_1ρ_ evaluation before and after the treatment.

	PRE		POST		
	MEAN	STD	MEAN	STD	*p*-Value
**P_1**	45.39	4.09	59.42	1.99	0.0005
**P_2**	44.84	2.54	53.36	6.97	0.0079
**P_3**	49.9	2	53.4	7	0.1797
**P_4**	72.16	2.86	53.36	6.72	0.00058

## References

[1] Arendt-Nielsen L., Graven-Nielsen T., Graven-Nielsen T., Arendt-Nielsen L., Mense S. (2008). Translational aspects of musculoskeletal pain: From animals to patients. Fundamentals of Musculoskeletal Pain.

[2] Bove S.E., Flatters S.J., Inglis J.J., Mantyh P.W. (2009). New advances in musculoskeletal pain. Brain Res. Rev..

[3] Sinatra R. (2010). Causes and consequences of inadequate management of acute pain. Pain Med..

[4] Clauw D.J. (2015). Diagnosing and treating chronic musculoskeletal pain based on the underlying mechanism(s). Best Pract. Res. Clin. Rheumatol..

[5] Blyth F.M., Noguchi N. (2017). Chronic musculoskeletal pain and its impact on older people. Best Pract. Res. Clin. Rheumatol..

[6] Bartels E.M., Danneskiold-Samsøe B. (1986). Histological abnormalities in muscle from patients with certain types of fibrositis. Lancet.

[7] Sharafi A., Chang G., Regatte R.R. (2017). Bi-component T1rho and T2 Relaxation Mapping of Skeletal Muscle *In-Vivo*. Sci. Rep..

[8] Stevens K.J., McNally E.G. (2010). Magnetic resonance imaging of the elbow in athletes. Clin. Sports Med..

[9] Walz D.M., Newman J.S., Konin G.P., Ross G. (2010). Epicondylitis: Pathogenesis, imaging, and treatment. Radiographics.

[10] Menon R.G., Raghavan P., Regatte R.R. (2019). Quantifying muscle glycosaminoglycan levels in patients with post-stroke muscle stiffness using T(1ρ) MRI. Sci. Rep..

[11] Gilani I.A., Sepponen R. (2016). Quantitative rotating frame relaxometry methods in MRI. NMR Biomed..

[12] Mlynarik V., Szomolanyi P., Toffanin R., Vittur F., Trattnig S. (2004). Transverse relaxation mechanisms in articular cartilage. J. Magn. Reson..

[13] Peng X.G., Wang Y.C., Zhang S.J., Bai Y.Y., Mao H., Teng G.J., Ju S.H. (2017). Noninvasive assessment of age, gender, and exercise effects on skeletal muscle: Initial experience with T1 rho MRI of calf muscle. JMRI.

[14] Paul C.P.L., Smit T.H., De Graaf M., Holewijn R.M., Bisschop A., Van De Ven P.M., Mullender M.G., Helder M.N., Strijkers G.J. (2018). Quantitative MRI in early intervertebral disc degeneration: T1rho correlates better than T2 and ADC with biomechanics, histology and matrix content. PLoS ONE.

[15] Luomala T., Pihlman M., Heiskanen J., Stecco C. (2014). Case study: Could ultrasound and elastography visualized densified areas inside the deep fascia?. J. Bodyw. Mov. Ther..

[16] Stecco A., Meneghini A., Stern R., Stecco C., Imamura M. (2014). Ultrasonography in myofascial neck pain: Randomized clinical trial for diagnosis and follow-up. Surg. Radiol. Anat..

[17] Mense S., Hoheisel U. (2016). Evidence for the existence of nociceptors in rat thoracolumbar fascia. J. Bodyw. Mov. Ther..

[18] Yahia L., Rhalmi S., Newman N., Isler M. (1992). Sensory innervation of human thoracolumbar fascia. An immunohistochemical study. Acta Orthop. Scand..

[19] Stecco C., Stern R., Porzionato A., Macchi V., Masiero S., Stecco A., De Caro R. (2011). Hyaluronan within fascia in the etiology of myofascial pain. Surg. Radiol. Anat..

[20] Matteini P., Dei L., Carretti E., Volpi N., Goti A., Pini R. (2009). Structural behavior of highly concentrated hyaluronan. Biomacromolecules.

[21] Pintucci M., Simis M., Imamura M., Pratelli E., Stecco A., Ozcakar L., Battistella L.R. (2017). Successful treatment of rotator cuff tear using Fascial Manipulation(^®^) in a stroke patient. J. Bodyw. Mov. Ther..

[22] Cotti A., Del Corso M., Diana R., Cornale L., Sudanese A., Stecco A., Branchini M. (2019). Inter and Intra Operator Reliability of Motor and Palpation Evaluation in Fascial Manipulation in individuals with coxarthrosis. J. Man. Manip. Ther..

[23] Cowman M.K., Schmidt T.A., Raghavan P., Stecco A. (2015). Viscoelastic Properties of Hyaluronan in Physiological Conditions. F1000Research.

[24] Mense S. (1993). Nociception from skeletal muscle in relation to clinical muscle pain. Pain.

[25] Song Z., Banks R.W., Bewick G.S. (2015). Modelling the mechanoreceptor’s dynamic behaviour. J. Anat..

[26] Todd A.J. (2010). Neuronal circuitry for pain processing in the dorsal horn. Nat Rev Neurosci..

[27] Wenzke D.R. (2013). MR imaging of the elbow in the injured athlete. Radiol. Clin. N. Am..

[28] Stecco A., Gesi M., Stecco C., Stern R. (2013). Fascial components of the myofascial pain syndrome. Curr. Pain Headache Rep..

[29] Stecco C., Fede C., Macchi V., Porzionato A., Petrelli L., Biz C., Stern R., De Caro R. (2018). The fasciacytes: A new cell devoted to fascial gliding regulation. Clin. Anat..

[30] Phillips K., Clauw D.J. (2011). Central pain mechanisms in chronic pain states - Maybe it is all in their head. Best Pract. Res. Clin. Rheumatol..

[31] Sarzi-Puttini P., Atzeni F., Mease P.J. (2011). Chronic widespread pain: From peripheral to central evolution. Best Pract. Res. Clin. Rheumatol..

